# Evaluation of the Dangerous Decibels Brazil Program in Workers Exposed to Noise

**DOI:** 10.3389/fnint.2022.909972

**Published:** 2022-07-14

**Authors:** Luciana Bramati, Jair Mendes Marques, Claudia Giglio Oliveira Gonçalves, David Welch, Ravi Reddy, Adriana Bender de Moreira Lacerda

**Affiliations:** ^1^Program in Communication Disorders, Tuiuti University of Paraná, Curitiba, Brazil; ^2^School of Population Health, University of Auckland, Auckland, New Zealand; ^3^Occupational Safety and Health, Massey University, Auckland, New Zealand; ^4^School of Speech Therapy and Audiology, University of Montreal, Montreal, QC, Canada

**Keywords:** hearing, noise-induced hearing loss, hearing protection, knowledge, habits, attitudes

## Abstract

**Introduction:**

Noise-induced hearing loss can be avoided by taking preventive measures.

**Objective:**

To assess the effectiveness of the Brazilian version of the Dangerous Decibels^®^ program for noise-exposed workers, using the ecological model as an educational intervention plan.

**Method:**

Non-randomized interventional study with a quantitative, experimental trial design, conducted at a meatpacking company. The participants were divided into two groups—the first one (*n* = 132, divided into 6 subgroups) received the Dangerous Decibels^®^ Brazil educational intervention (DDBEI) adapted to workers while the second group (*n* = 138, divided into 5 subgroups) received a conventional educational intervention (CEI). The interventions lasted 50 min. The Hearing Protection Assessment Questionnaire (HPA-5) was administered before and after the interventions. The five dimensions (attitude, behavior, knowledge, supports, and barriers) were compared using the Student’s *t*-test for paired data (<0.05).

**Results:**

After both the DDBEI and CEI training, workers improved significantly in barriers, supports, knowledge, attitudes, and behavior around noise. By chance, the CEI group scored lower in all measures than the DDBEI group before training, and though both groups improved, the difference was maintained after training.

**Conclusion:**

The Brazilian version of the Dangerous Decibels^®^ program for noise-exposed workers was effective, influencing positively the factors at different levels of the ecological model. Though the DDBEI was no more effective than the CEI, the CEI participants began at much lower levels, so the effectiveness of the DDBEI may have been underestimated.

## Introduction

Brazil has public and federal policies related to workers’ health, and their purpose is to define the principles, guidelines, and strategies to be observed by the three spheres of the Unified Health System for the development of comprehensive care to workers’ health, with emphasis on surveillance, promotion and protection of workers’ health and the reduction of morbidity and mortality resulting from development models and production processes (Brasil, 2012). The federal regulatory standards relating to occupational safety and medicine have mandatory compliance by private and public companies and public agencies of direct and indirect administration, as well as by agencies of the Legislative and Judiciary Branches, which have employees governed by the Consolidation of Labor Laws. These guide health actions, including auditory health actions, in work environments.^1^

Noise-Induced Hearing Loss (NIHL) is considered the most common health problem among workers in several industrial activities worldwide and can damage health and quality of life. However, NIHL can be avoided if preventive measures are adopted (Nelson et al., 2005; Sliwinska-Kowalska and Davis, 2012; Brasil, 2020).

Therefore, agencies recommend the implementation of Hearing Loss Prevention Programs (HLPP) in the work environment (NIOSH - National Institute for Occupational Safety and Health, 1996, 2018; OSHA - Occupational Safety and Health Administration, 2002; Brasil, 2020; Conselho Federal de Fonoaudiologia – CFFa, 2021). Educational interventions are an essential part of this program. They provide workers with the chance to rethink their health and quality of life and work, generating safer, and more stimulating working conditions (Oliveira et al., 2015; Kim et al., 2016; Ramos et al., 2017).

The Ecological Model for Health Promotion, which uses more than one behavior change theory targeting individual and environmental influences, is considered more effective in health promotion interventions (Kok et al., 2008; Sallis et al., 2008; Angus et al., 2013). This model provides an opportunity to identify gaps in NIHL prevention and develop educational interventions targeted at different levels of influence on hearing preservation behavior.

The Ecological Model for Health Promotion (Mcleroy et al., 1988) is an extension of Bronfenbrenner’s theory and is conceptualized by five social levels corresponding to Bronfenbrenner’s levels, which include: the intrapersonal level (the individual characteristics such as knowledge, attitudes, values, and skills), the interpersonal level (social relationships including family, peers, and peer networks), the organizational level (organizational norms, policies, and support), the community level (community norms, standards, and social media), and the policy level (health promotion policies and legislation and their regulation, interpretation, and enforcement).

The MATCH - Multi-level Approach to Community Health Model (Simons-Morton et al., 2012) ecological planning model was used to adapt a classroom hearing loss prevention program named Dangerous Decibels^®^ (DD) for use with workers (Reddy, 2014; Reddy et al., 2017). The DD program^2^ was originally developed and proven effective for children in schools in Oregon and Washington (Martin et al., 2006; Griest et al., 2007) and in other countries, including Brazil (Knobel and Lima, 2013). The DD mission is to significantly reduce the prevalence of noise-induced hearing loss and tinnitus through exhibits, education, and research. The goal of the program is to improve knowledge, attitudes and behaviors regarding noise exposure and hearing protection strategies (Martin et al., 2006).

The behavioral health education pedagogical design used in Dangerous Decibels^®^ prioritizes educational aspects linked to individual and environmental behavioral risk factors, using the health belief model, the social cognitive theory, and ecological model for health promotion as a pedagogical intervention plan. It proved effective for workers in New Zealand, promoting knowledge and change of habits, attitudes, and behaviors regarding noise and the use of hearing protection by workers (Reddy, 2014; Reddy et al., 2017). Thus, bringing a new perspective to educational interventions in the occupational context is an interactive and dynamic program that provides greater worker participation (Reddy, 2014).

There is no hearing health program for workers employing the behavioral pedagogical conception using the ecological model as a pedagogical intervention plan in Brazil. The implementation of a program using these principles would be a great contribution to the Brazilian worker. Instead, most Brazilian programs use traditional pedagogical conceptions for the educational interventions for workers. Considering the aspects addressed here, we propose to answer the following question: “Will the educational intervention proposed by the Dangerous Decibels Brazil (DDB) program prove effective when compared to conventional educational intervention?”

This study aims to evaluate the effectiveness of the educational intervention Dangerous Decibels Brazil for workers exposed to noise compared to the conventional educational intervention proposed by the company.

## Methodology

### Study Type and Location

The Ethics Committee of the Graduate Program in Communication Disorders at the Universidade Tuiuti do Paraná approved this study, process 2.725.935, and the company approved it. The study is a non-randomized interventional study of the experimental, quantitative type conducted in a meatpacking plant.

The company was selected because it is a local company, with its headquarters and most of its branches in the same city in the south of the country. It is part of a cooperative, being considered one of the largest food cooperatives in Brazil, formed by more than a hundred-thousand families, a total that includes forty thousand direct jobs, besides the 10,000 employees and the 65,000 families of rural entrepreneurs from the 11 cooperatives that are part of its system.

As it is a company that has always sought to invest in better health conditions for its employees, the company follows the recommendations of the federal government and has health programs described in the Regulatory Norms (RN), such as RN-6 on Individual Protection Equipment, RN-7 on the Occupational Health Medical Control Program, RN-15 on Unhealthy Activities and Operations, RN-17 on Ergonomics, RN-36 on Safety and Health at Work in slaughterhouses and meat and meat processing companies. It develops actions aimed at minimizing the risks caused by noise, which ranges from 78 to 120 dB HL (depending on the sectors and locations), through improvements in the work environment, use of hearing protection equipment and awareness of its workers. The company also has an auditory conservation program.

### Selection, Inclusion, and Exclusion Criteria—Participants

The sample selected was by convenience, as the researcher had access to the location and participants. This study’s participants were selected during the admission selection process and invited to participate in the survey at the integration process held at the company. Initially, the number of participants was 509. However, during the 3-month interval, the intervention period, 239 participants did not remain in the company. Therefore, 270 Southern Brazilian workers of both genders participated in the study, distributed into the DDB experimental/intervention group (DDBEI: *n* = 132) and the conventional control/intervention group (CEI: *n* = 138).

### Instruments

We used as instruments: (a) the Dangerous Decibels Brazil educational intervention for workers (DDBEI) and the company’s conventional educational intervention (CEI); and (b) the Hearing Protection Assessment Questionnaire (HPA-5). The current study replicates the original New Zealand research that used the validated HPA-5 Questionnaire as the data collection tool (Reddy, 2014; Reddy et al., 2017). The hearing protection assessment questionnaire assessing five measures (HPA-5) is an extension of the two-measure (HPA-2) questionnaire developed and described elsewhere (Reddy et al., 2014). The HPA-5 assessed barriers and supports, knowledge, attitudes and behavioral measures toward hearing protection. The knowledge, attitudes and behavioral measures were adapted from a questionnaire used to assess the effectiveness of the school-based Dangerous Decibels Programme in the United States of America (Griest et al., 2007). The Hearing Protection Assessment Questionnaire (HPA-5) was translated and adapted to Portuguese, named *Questionário de Avaliação da Proteção Auditiva* (APA; Supplementary Appendix) by Bramati et al. (2021) (in press, Codas, 2022), applied to both groups before and after the educational intervention.

Educational Interventions for Workers - At this stage (3 months after the admission exam), the participants were randomly divided into two groups, where the first group received the DDBEI adapted for workers (Reddy et al., 2017) and provided by the researcher Speech Therapist, Dangerous Decibels Brazil Educator and the other half received the traditional educational intervention (TEI), provided by the health and safety team.

The DDBEI was conducted at the company’s premises in a group of 132 workers, divided into 6 groups with an average of 22 workers in each group. The EIDDB intervention lasted 50 min.

The DDBEI was inspired by Reddy (2014) and Reddy et al. (2017) and reinforced key messages to improve and motivate hearing health behaviors in workers. All modules used different strategies, such as demonstrations, audio-visual resources, use of objects, worker involvement, and interaction, to convey the program’s messages. It was essential to the program’s objectives, especially when effective training involves strategies such as:

(a) delivery of relevant information and concepts;
(b) demonstration of knowledge, attitudes, and skills to be taught;
(c) opportunity to practice the skills learned; and
(d) facilitation of feedback between the educator and the learner/participant (Salas and Cannon-Bowers, 2001).

**FIGURE 1:**
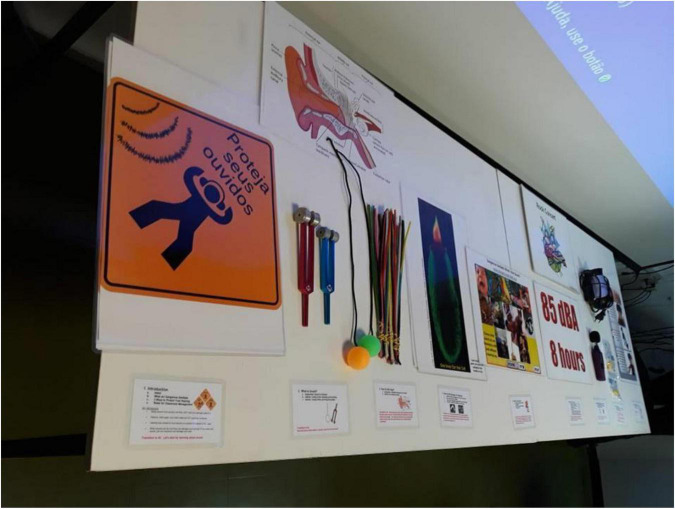
Summary version of the script DDBEI and educational materials.

This study used the Dangerous Decibels^®^ program training script (manual) developed by Reddy (2014) and Reddy et al. (2017) before the program was conducted at workplaces. It comprises completing systematic training instructions on approaching each of the program’s components and how to carry them out. In addition, the script encourages educators to include or generate discussions on examples relevant to the training participants. Furthermore, a summary version of the script was developed as a series of nine cards for each module. Figure 1—DDBEI.

The DDBEI was conducted after cultural adaptation for Brazilian workers using mainly examples and situations that describe the work reality within the company, and was divided into nine modules proposed by Reddy (2014) and Reddy et al. (2017).

*Module 1: Program objective and introduction*:

The workplace DDBEI included more occupational sector-specific information than the original school-based Dangerous Decibels^®^ program, such as the high prevalence of NIHL affecting workers and increasing economic and social costs. In addition, there was more emphasis on workplace noise control strategies, such as engineering measures, administrative measures, and individual hearing protection.

In addition to the original Dangerous Decibels^®^ program messages of “stay away,” “protect your ears” and “turn down the volume,” messages in the occupational context such as “eliminate,” “isolate” “minimize” were emphasized as warning signs and signs on the dangerous level of noise sources were displayed to communicate these messages.

*Module 2: The physics of sound and energy (sound/energy)*:

This part of the original Dangerous Decibels^®^ program was fully maintained from the school-based program to the workplace version. The objective was to involve the workers and give concrete examples that would help them understand the concept of sound energy as something that can cause harm.

*Module 3: Ear*:

An ear anatomy poster was used to explain how sound waves reach the ear and provide a basic explanation of the processes occurring to make sound heard. It included understanding the physiology of auditory sensory cells (hair cells) and sound detection at a basic level. This explanation of a complicated concept in a simple, concrete form facilitated understanding.

*Module 4: The hearing loss process (hearing damage)*:

This module demonstrates how high sound pressure levels damage the ear’s hair cells. This part was based on the previous modules, describing vibrations and how the hair cells are involved in the hearing process. In addition, it helped reinforce the messages regarding the susceptibility and severity of noise dangerous to human hearing.

*Module 5: The hearing loss consequences (experience/hearing loss)*:

Hearing loss simulation software (Huckvale, 2010) was used to demonstrate the hearing loss effects. The module emotionally and reflexively emphasized the consequences of hearing loss and its effect on life quality. Workers were encouraged to discuss how they spent time with family and friends, and the simulator was used to demonstrate how Hearing Loss can affect activities and social interactions.

*Module 6: Workplace sounds loudness (sound sources/flashcards)*:

The decibel scale was introduced with an emphasis on the 85 dB tolerance limit. We also discussed the concept of reducing exposure time when noise levels increase. Workers were encouraged to engage in an activity involving several *flashcards* with images of common work tools and activities. The DDBEI included examples specific to the occupational context, such as power tools and heavy machinery, along with other examples such as tractor noise, washing machine noise, and rock concerts.

*Module 7: Sound measurement (experience/distance sound pressure levels)*:

The workers learned how to measure sound using a sound pressure level meter. Next, a drill was used as a sound source to demonstrate the noise level. Then, the concept of reducing noise exposure by moving away from the sound source was demonstrated and discussed. In addition, there was a discussion regarding machines creating different noise levels when applied to different materials such as wood, glass, or steel.

*Module 8: Proper use and maintenance of hearing protection devices (HPD)*:

The correct method for inserting hearing protectors and ensuring adequate protection was demonstrated. The workers were encouraged to practice the correct procedure with their fellow workers. The DDBEI also emphasized the importance of correctly wearing protectors with caps and/or long hair. HPD maintenance was discussed, and workers were encouraged to seek management assistance to ensure a high HPD standard. The objective was to improve the workers’ self-efficacy.

*Module 9: Peer modeling and workplace hearing health promotion (experience/work environment)*:

The DDBEI used this component to encourage peer modeling and promote hearing health in their settings. The emphasis was on creating a working environment that takes hearing health promotion seriously. For example, the classroom program explores the hearing protection behavior of children and their friends when exposed to high noise levels during rock concerts. In addition, the work program was adapted to encourage the worker regarding their own and their colleagues’ hearing protection behavior when exposed to workplace noise.

The CEI was conducted on the company’s premises, with a group of 138 workers, divided into 6 groups with an average of 23 workers in each group. The CEI lasted 50 min. It was divided into 5 modules and was performed on a single day. The CEI was carried out with a slide presentation, where aspects regarding hearing protection care were addressed:

*Module 1: Program objective and introduction*:

The CEI in the workplace and occupational sector-specific information relays the program’s objectives, providing information on the high prevalence of NIHL affecting workers and increasing economic and social costs. In addition, there was more emphasis on workplace noise control strategies, such as engineering measures, administrative measures, and individual hearing protection.

*Module 2: NIHL—Hearing Anatomy and Physiology*:

This module explains how the auditory system works, using visual resources to explain the subject.

*Module 3: Noise—concept and characteristics*:

The decibel meter instrument was used to demonstrate the noise levels at different locations in the room, explaining its concept and characteristics.

*Module 4: HLPP—Hearing Loss Prevention Program*:

An oral explanation explained how the hearing loss prevention program is developed within the company, which laws refer to this program, and what role each participant should play.

*Module 5: How to prevent NIHL*:

In this module, the participants received information through oral explanations and visual resources on measures to reduce noise levels in the workplace, the importance of wearing hearing protectors, and awareness of the importance of each person’s role in decreasing noise levels.

(b) HPA-5 Questionnaire: The Brazilian version of the HPA-5 questionnaire named *Avaliação da Proteção Auditiva* (APA) (Bramati et al., 2021, in press, Codas) was used before and after the educational intervention (DDBEI and CEI). The APA was applied to evaluate the DDBEI’s effectiveness. The APA questionnaire was applied to all workers who took the admission exam (audiometry) and after, immediately after participating in the educational intervention (DDBEI and CEI). The APA assessed barriers and supports, knowledge, attitudes, and behavioral measures regarding hearing protection. Knowledge, attitudes, and behavioral measures were adapted from a questionnaire used to assess the effectiveness of the school-based Dangerous Decibels^®^ Program in the United States (Griest et al., 2007). The scales related to knowledge, attitudes, and behavior have multiple choice questions, each of which has only one correct answer. There are five questions for the knowledge scale about sound science, hearing loss, and hearing conservation, two questions related to the attitudes measure about noise protection and hearing protection two questions about work safety behavior attitudes (questions 7 and 8), and three questions about behavior (questions 10, 20, and 21). The measures regarding barriers and supports included nine items, each describing why they (support) and would not wear (barriers) HPD when exposed to noise at work. It allowed respondents to endorse any item they identified with for each measure. The two questions related to Support are questions 9 and 11. Question 11 has four subscales in the responses (safety culture, risk recognition, behavior motivation, and safety culture). The Barriers-related question is question 12, with two subscales in the responses (justification of risk and restrictions on DPA use).

The questionnaire also included demographic items, such as gender and age. In addition, two items describe attitudes toward safety behavior at work, and one item documents HPD self-reported use.

### Data Analysis

Comparisons were made separately for the five scales (attitude, behavior, knowledge, supports, and barriers) assessed using Student’s *t*-test for paired data to detect significant differences in results between pre-intervention and post-intervention. All tests were considered at the 0.05 significance level.

Considering that the five scales evaluated in the pre- and post-intervention questionnaire have different numbers of items, the response scores were converted into percentages to allow comparability among them, and for attitude, behavior, and knowledge, into hit percentages, where the analysis form recommended for the Dangerous Decibels^®^ program was followed. In addition, the percentage of marked items was considered for supports and barriers since the answers for these scales were presented as affirmative sentences.

The independent variables were Time, which had two levels (pre-training and post-training), and Training method, which also had two levels (DDBEI and CEI). In each model, training type (DDBEI or CEI) was a between-subjects factor, and the two measures (pre- and post-training) were treated as repeated measures.

Five repeated measures analyses of variance (ANOVAs) were conducted to test training effects on the five scales. The five outcome measures were: knowledge, attitudes, behavior, supports, and barriers. Each outcome measure was modeled with a separate ANOVA procedure and treated as a repeated measure over time, while the training groups were treated as independent. The interaction between Time and Training was used to test the hypothesis that the training methods differed in effectiveness. If the interaction were significant, it would mean that the outcome measure for one training group changed more than the same measure for the other training group. The homoscedasticity and normality assumptions were graphically examined for the change (from pre- to post-training) in the five scales, and all were satisfactory. We used a 0.05 alpha criterion level.

The data were verified for statistical test assumptions. Given that the repeated measures approach was used, the change in scores between pre- and post-training measures was evaluated, and visual inspection of the histogram showed approximately normal distributions. There were three scores on the knowledge scale (two in the DDBEI group and one in the CEI group) where participants scored lower after the intervention than before. However, removing these cases from the analysis did not affect the findings, so they were left for the results presented.

## Results

Table 1 presents the results of the participants’ profiles according to the variables gender, sector, shift, position, and nationality.

**TABLE 1:** Participants’ profile in the CEI group (*n* = 132) × DDBEI group (*n* = 138).

Variable	DDBEI group	CEI group	*p*
Gender	*n* (%)	*n* (%)	
Female	77 (58.3)	75 (54.3)	0.5083
Male	55 (41.7)	64 (45.7)	0.5083
**Sector/average NPS**			
Cutting (A B C)/89.8	103 (78.0)	104 (75.4)	0.3068
Packaging (A B)/91.6	15 (11.4)	19 (13.8)	0.2764
Scalding A/94.4	1 (0.8)	(0.8)	NA
Evisceration (A B)/89.1	10 (7.6)	8 (5.8)	0.5542
Sanitation C/91.2	3 (2.3)	4 (2.9)	NA
Tunnels A/76.9	– (0.0)	2 (1.4)	NA
**Shift**			
First	49 (37.1)	54 (39.1)	0.7263
Second	66 (50.0)	60 (43.5)	0.2689
Third	17 (12.9)	24 (17.4)	0.2852
**Position/function**			
Production operator I	96 (72.7)	83 (60.1)	0.0237*
Production operator II	30 (22.7)	40 (29.0)	0.2211
Production operator III	3 (2.3)	10 (7.2)	NA
Sanitizer I	3 (2.3)	3 (2.2)	NA
Production balancer	– (0.0)	2 (1.4)	NA
**Country of birth**			
Brazil	119 (90.2)	121 (87.7)	0.4972
Haiti	13 (9.8)	17 (12.3)	0.5973

The Test for Difference of Proportions was applied at a 0.05 significance level. NA, the test is Not Applicable. * significant difference.

### Dangerous Decibels^®^ Brazil Educational Intervention Results

**FIGURE 2:**
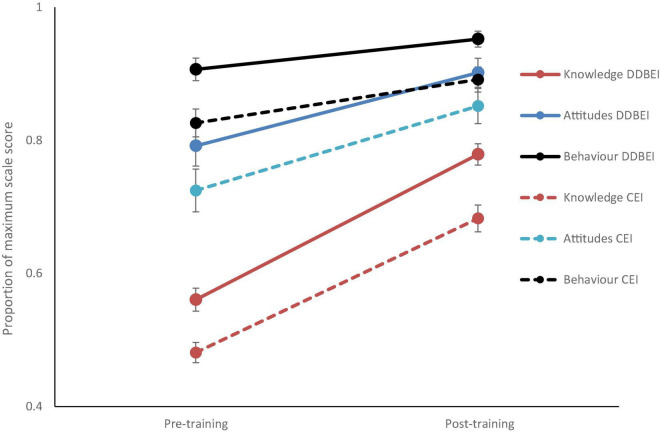
Graph of mean Knowledge, Attitude and Behavior scores as a proportion of the maximum possible on each scale, before and after training with the Dangerous Decibels (DDBEI) and Conventional (CEI) training methods. Error bars represent one standard error of the mean.

Figure 2 presents the scale results for attitudes, behaviors, and knowledge in the DDBEI pre- and post-intervention questionnaire. Significant increases were observed after the intervention for all scales at *p* < 0.001.

**FIGURE 3:**
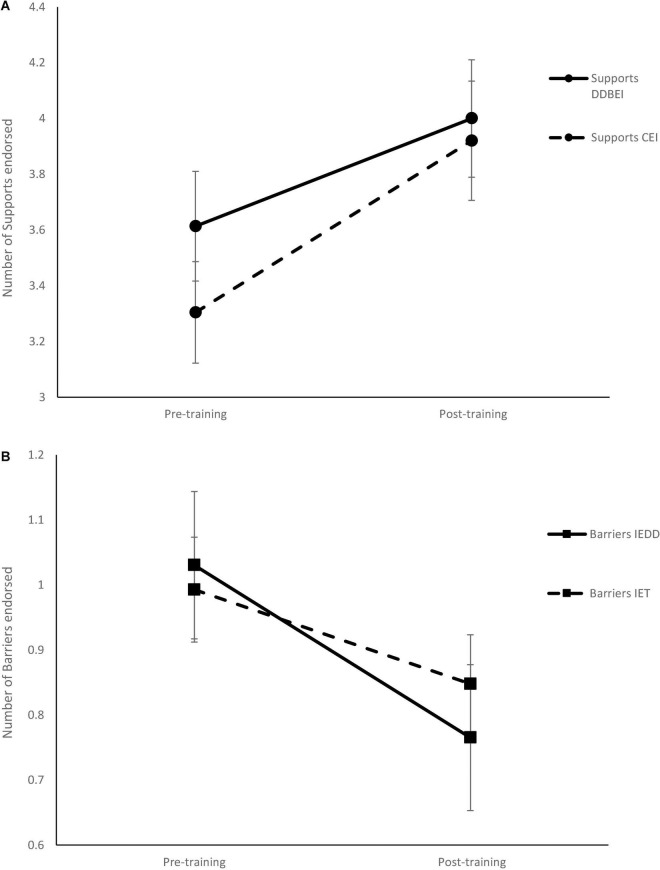
Change in the number of Supports **(A)** and Barriers **(B)** for hearing-protective behavior before and after training with the Dangerous Decibels (DDBEI) and Conventional (CEI) training methods. Error bars represent one standard error of the mean.

Figure 3 presents the scale results for supports and barriers in the DDBEI pre- and post-intervention questionnaire. Significant improvements were observed pre- and post-intervention for both scales at *p* < 0.001.

### Conventional Educational Intervention Results

Figure 2 presents the scale results for attitudes, behaviors, and knowledge in the CEI pre- and post-intervention questionnaire. Again, significant improvements were observed before and after the intervention for all scales at *p* < 0.001.

Figure 3 presents the scale results for supports and barriers in the CEI pre- and post-intervention questionnaire. Again, significant improvements were observed pre- and post-intervention for both scales at *p* < 0.001.

### Comparison Between Dangerous Decibels^®^ Brazil Educational Intervention and Conventional Educational Intervention Interventions

Figure 2 presents the DDBEI and CEI comparisons on the attitudes, behaviors, and knowledge scales.

Figure 3 presents the comparison for supports and barriers in the pre- and post-intervention questionnaire in the DDBEI and CEI groups.

The overall effects showed an increase in all five scales after the intervention for both groups, implying that the DDBEI and CEI methods were both effective [*F*(1, 268) = 179.313, *p* < 0.001]. However, there was a pre-existing difference between the two intervention groups in which the group receiving the DDBEI scored higher on all measures (and lower on Barriers) before the intervention. Therefore, statistical tests compared the overall effects, where we observed a difference between the groups (DDBEI and CEI). However, this difference was present before and after the intervention.

No interaction between time and training was found for any of the five scales [*F*(1, 268) = 0.285, *p* = 0.594]. This means that both groups improved pre- and post-intervention similarly. Thus, both DDBEI and CEI were effective and caused equal improvement after the intervention.

## Discussion

The pedagogical conception of the behavioral type in DDBEI and the use of the ecological model to identify and direct hearing preservation behavior at different levels of influence contributed to the Brazilian workers’ reflection on the preservation of their hearing and health when exposed to noise. The results showed that the DDBEI effectively improved several measures that positively influenced the wearing of hearing protection devices in workers. These results align with Reddy et al. (2017), where their results show a significant effect of the intervention measures over time, indicating that these measures improved significantly after the intervention.

This study observed improved motivation for healthy behaviors and habits and increased knowledge. This data is especially important since workers new to the company tend to model their protective behavior based on the behaviors of more experienced workers. Moreover, according to the Social Cognitive Theory principles, behavior is initiated, and maintained by the reciprocal influences between the person, the behavior, and the environment (Bandura, 1986). Therefore, interventions employing active training methods are more effective in reducing negative health outcomes and promoting worker safety and health (Burke et al., 2006). According to the ecological model, intrapersonal and interpersonal influences strengthen organizational norms and culture at the organizational level that supports health promotion (Reddy, 2014).

We observed significant pre- and post-intervention differences regarding supports, demonstrating an increase in the aspects supporting the proper use of HPD. Risk recognition, behavioral motivation, and company safety culture are important aspects to consider as support and include the influences of peer modeling on hearing protection behavior at the interpersonal level. At the organizational level, employer modeling, workplace rules compliance, and training influence motivation and safety culture. These results support evidence that workers’ acceptance and promotion of workplace safety and protective behavior is an HDP predictor (Edelson et al., 2009).

According to Areosa (2007), regarding risk perceptions at work, they are constructed by multiple factors, knowing that they can have a diversified capacity to influence the worker. We find that the risk perception at work is a variable phenomenon within the set of social actors. For example, a given factor can exert an extraordinary influence on one individual’s behaviors, attitudes, and representations and be indifferent to another. In part, this ambiguity characterizes risk perceptions at work. Thus, heterogeneity, ambivalence, and uncertainty characterize risk perception at work. Areosa (2012) found that workers’ risk perceptions in the early days at a job position may correspond to a greater perception of occupational hazards, if we consider that they make more use of HPD. It is pertinent to remember that workers’ risk perceptions are absolutely “real and objective” for them, and they tend to act upon those perceptions. Therefore, integrating the different risk perceptions of workers into risk analyses is a key step toward the success of an organizational risk management program and, consequently, toward accident prevention.

Regarding the barriers related to restrictions on the use of hearing protection, we observed significant differences pre- and post-intervention, showing a decrease in barriers. It corroborates Reddy (2014), where the results show a significant intervention effect on the use of hearing protection over time, with a 26% improvement in the consistent use of hearing protection, and 44% of workers in the group reporting always using hearing protection when exposed to noise before the intervention. After the intervention and at the 8-week follow-up, 70% of the workers reported always wearing hearing protection when exposed to noise.

When comparing the DDBEI with the CEI, we observed no significant differences between the interventions. However, the workers showed significant improvements on all five scales after the two interventions. This finding applies to attitudes, knowledge, and behavior, similarly, to supports and barriers. It is worth noting that the CEI group scored lower on the scales than the pre-intervention DDBEI group. It was unexpected, and it is possible that if the two groups were homogeneous, we would have observed a difference in the result. For example, it is possible that the effectiveness of the DDBEI was concealed by the higher pre-training level of that group compared to the group trained with the CEI.

However, considering that the DDBEI was new to the company’s workers who were used to passively participating in traditional educational interventions, the DDBEI was well received and accepted by the Brazilian workers and their managers. They appreciated the opportunity, the relevance, and the modules’ content. It suggests that this program does not disrupt workplace practices and encourages hearing health promotion and the prevention culture.

The prevention culture concept is implicitly based on the safety culture concept (HSE - Health and Safety Executive, 2005). Both use a cultural approach. A safety culture aims to reduce work-related risks, while a prevention culture aims to reduce both work-related and non-work-related risks. Safety culture is mainly directed at the workplace level, while prevention culture is directed at the societal or national level. In a safety culture, the emphasis is on health protection, while in prevention culture, it emphasizes health protection and promotion (ILO–International Labour Organization, 2014; European Commission, 2020). Most probably, the practical nature of the educational intervention helped workers understand the hearing health concepts as a relevant issue (Reddy et al., 2017).

### Limitations

The study had limitations. This study was conducted with a Southern Brazilian convenience sample of workers from a meatpacking plant, not representing all Brazilian workers, making it necessary to evaluate DDBEI in other country regions. The project was conditioned to 1 year, being possible to apply the questionnaires before and after the intervention, not being possible to evaluate the follow-up after 6 months or 1 year. Finally, another factor considered important was the difference between the groups in the pre-intervention, with the DDBEI group showing a higher score on all scales. Perhaps it would be possible to identify differences between the groups if it did not happen.

### Recommendations for Future Studies

We suggest applying the questionnaire at four time-points: pre-intervention, post-intervention, and at 3- and 6-month periods, so the results can be observed over time. We also suggest comparing the intervention in homogeneous groups since the pre-intervention and improving the program with educational strategies, focusing on the risk justification subscales and restrictions on the HPD use regarding the barriers scale. In addition, on the safety culture, risk recognition, and behavior motivation subscales, relating to the supports scale, so that significant results on these scales can be observed in further studies.

The survey focused primarily on three ecological model levels: intrapersonal, interpersonal, and organizational. However, there is room for research and the development of interventions targeting the community as a whole, directing future research in this scenario.

## Conclusion

When comparing the DDBEI with the CEI, we observed no significant differences between the interventions. However, the DDBEI for workers exposed to noise in occupational settings proved effective and contributed to worker training by increasing knowledge, changing attitudes, and intrapersonal behavior, while also increasing support and reducing barriers regarding HPD use. Furthermore, the results obtained with the DDBEI for workers will contribute to developing new proposals and materials specific to the DDB program, targeted to be offered as another alternative to the NIHL Prevention Programs.

## Data Availability Statement

The raw data supporting the conclusions of this article will be made available by the authors, without undue reservation.

## Ethics Statement

The studies involving human participants were reviewed and approved by the Ethics Committee of the Graduate Program in Communication Disorders at the Universidade Tuiuti do Paraná approved this study, process 2.725.935. The patients/participants provided their written informed consent to participate in this study. Written informed consent was obtained from the individual(s) for the publication of any potentially identifiable images or data included in this article.

## Author Contributions

AL and LB: conceptualization, data curation, investigation, and writing—original draft preparation. AL, LB, DW, and RR: methodology. CG and JM: validation. JM and DW: formal analysis. AL, LB, CG, RR, and DW: writing—review and editing. All authors contributed to the article and approved the submitted version.

## Conflict of Interest

The authors declare that the research was conducted in the absence of any commercial or financial relationships that could be construed as a potential conflict of interest.

## Publisher’s Note

All claims expressed in this article are solely those of the authors and do not necessarily represent those of their affiliated organizations, or those of the publisher, the editors and the reviewers. Any product that may be evaluated in this article, or claim that may be made by its manufacturer, is not guaranteed or endorsed by the publisher.

## Footnotes

1
www.gov.br/trabalho-e-previdencia/pt-br/composicao/orgaos-especificos/secretaria-de-trabalho/inspecao/seguranca-e-saude-no-trabalho/ctpp-nrs/normas-regulamentadoras-nrs

2
http://dangerousdecibels.org/
