# Supplementary material for: Evaluation of the Dangerous Decibels Brazil Program in Workers Exposed to Noise
Source: Front Integr Neurosci. 2022 Jul 14;16:909972. doi: 10.3389/fnint.2022.909972 (PMC9335485; doi:10.3389/fnint.2022.909972)
Supplement: Supplementary file 1 [file Data_Sheet_1.docx]

Supplementary material : Evaluation of the Dangerous Decibels Brazil (DDB) Program in Workers Exposed to Noise

**Appendix 1 -** *Questionário de Avaliação da Proteção Auditiva* (APA) – portuguese version

Como responder : preencha a sua resposta com um **X** ou escreva as suas respostas nas linhas.

**1**. **Gênero :** Masculino □ Feminino □

**2.** **Idade :** _____________anos Data de Nascimento : _____/_____/_____

**3**. **Cargo :** _______________________________ Turno : ________________

**4**. **A qual grupo étnico você pertence?** __________________________________

**5. Qual é seu país de nascimento?**

Brasil □ outro, por favor, especifique : _____________________________

**6. Se você respondeu « outro » para a questão 5, há quantos anos você está no Brasil?** ______

**7. Por favor, leia as duas frases cuidadosamente e escolha aquela que é mais verdadeira para você. Por favor, escolha A ou B :**

A. Para mim, a segurança está em primeiro lugar quando eu trabalho.

B. Para mim, a segurança é importante, mas outros fatores ou condições de trabalho, às vezes limitam à minha maneira de trabalhar de forma segura.

**8. Por favor, leia as duas frases cuidadosamente e escolha aquela que é mais verdadeira para você. Por favor, escolha A ou B :**

A. Os danos ocorrem no trabalho porque as pessoas não têm interesse suficiente em segurança

B. Os danos sempre ocorrerão no trabalho, não importa o quanto as pessoas tentem preveni-los

**9. Eu recebo protetores auditivos disponíveis para usar no meu trabalho.**

Sim □ Não □

**10. Eu uso protetores auditivos quando tem ruído no trabalho (por favor, assinale uma das opções abaixo) :**

Sempre □ Quase sempre □ Geralmente □ Às vezes □ Raramente ou Nunca □

**11. Se você usa protetores auditivos no trabalho, é porque : (por favor, marque todas aquelas que se aplicam) :**

A. Seu chefe diz para você fazer

B. Você está fazendo um trabalho ruidoso (ex. : trabalhando em máquinas ruidosas, com estrondos, pancadas, marteladas, etc.)

C. Outros trabalhadores estão fazendo tarefas ruidosas (ex. : trabalhando em máquinas ruidosas, com estrondos, pancadas, marteladas, etc.)

D. Você quer proteger sua audição

E. Você fica incomodado com o ruído

F. Você quer que sua audição esteja preservada para viver com qualidade junto à sua família

G. Seus colegas de trabalho te lembram de usá-los

H. São regras da sua empresa

I. Você recebeu treinamento para usá-los

Outro, por favor, especifique : ___________________________________________

**12. Se você não usa protetores auditivos quando está exposto ao ruído, é porque :** **(por favor, marque todas aquelas que se aplicam) :**

A. Não está claro para você quando você deveria usá-los

B. Você não consegue ouvir adequadamente para fazer seu trabalho (ex. Sinais de aviso, performance de máquinas)

C. Você não consegue se comunicar adequadamente com outros trabalhadores

D. Eles são desconfortáveis

E. Eles atrapalham o uso de outros equipamentos de segurança

F. Você está acostumado com o ruído no trabalho

G. Seus colegas frequentemente não o usam

H. Seus colegas acham engraçado quando você os usa

I. Outras pessoas também fazem tarefas ruidosas sem aviso

J. Outros, por favor, especifique :____________________________________________

**13. Perda auditiva pode ser curada com o uso de aparelhos auditivos (Selecione apenas um);**

**Verdadeiro □ Falso □ Não tenho certeza □**

**14. Medidas de som de _______________ e acima podem prejudicar a audição humana. (selecione apenas uma**) :

65 decibels (dBA) □ 70 decibels (dBA) □

85 decibels (dBA) □ Nenhuma destas alternativas □

**15. Sons que são muito altos podem prejudicar __________________, causando perda auditiva (assinale a alternativa que melhor preenche a frase acima e selecione uma apenas) :**


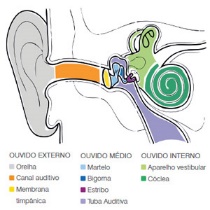


Canal auditivo □ Tímpano ou membrana timpânica □

Células ciliadas da cóclea □ Todas as opções acima □

**16.** **Perda auditiva causada por sons altos/ruídos é algo que pessoas ___________________** **podem ter (assinale a alternativa que melhor preenche a frase acima e selecione apenas uma) :**

Acima dos 60 anos □ Acima dos 40 anos □

Acima dos 50 anos □ Em qualquer idade □

**17. Posso proteger a minha audição de ruídos no trabalho usando protetores auditivos. (selecione apenas uma) :**

A. A todo momento quando está ruidoso

B. Apenas quando outras pessoas estão usando os seus

C. Apenas quando o ruído me incomoda

D. Apenas quando estou fazendo um trabalho ruidoso

E. Apenas quando meu chefe me orienta a usar

**18. Adquirir uma perda auditiva não é um grande problema para mim (selecione apenas uma):**

Concordo □ Discordo □ Não tenho certeza □

**19. Trabalhadores que estão expostos a sons altos/ruídos o tempo todo não parecem ter perda auditiva, assim, eu não tenho que me preocupar se tiver perda auditiva (selecione apenas uma);**

Concordo □ Discordo □ Não tenho certeza □

**20. Se o ambiente de trabalho estiver ruidoso, e meus colegas de trabalho não estiverem usando protetores auditivos (selecione apenas uma):**

A. Eu prossigo com meu trabalho e os deixo faz erem o que eles quiserem

B. Eu lembro e incentivo meus colegas a usarem seus protetores auditivos

C. Eu também tiro os meus porque eles não estão usando os deles

**21. Durante a semana passada, eu estive exposto a ruídos no trabalho sem usar proteção auditiva (selecione apenas uma):**

Sim □ Não □

**22. Uso protetores auditivos q uando outros trabalhadores estão fazendo um serviço ruidoso no trabalho.**

Sempre □ Quase sempre □ Geralmente □ Às vezes □ Raramente ou Nunca □

**23. Com que frequência seus colegas de trabalho usam protetores auditivos quando o ambiente de trabalho está ruidoso? (Selecione apenas uma):**

Sempre □ Quase sempre □ Geralmente □ Às vezes □ Raramente ou Nunca □
